# Effectiveness of the Temporal Flap in Reconstruction After Advanced External Ear Tumor Resection: A Case Report

**DOI:** 10.3390/clinpract16020027

**Published:** 2026-01-28

**Authors:** Kostadin Gigov, Petra Kavradzhieva, Ivan Ginev, Mihaela Bogdanova

**Affiliations:** 1 Section of Plastic Reconstructive and Aesthetic Surgery and Thermal Trauma, Department of Propedeutics of Surgical Diseases, Faculty of Medicine, “Saint. George” University Hospital Plovdiv, Medical University of Plovdiv, “Peshtersko shausse Blvd 66”, 4002 Plovdiv, Bulgaria; kostadin.gigov@phd.mu-plovdiv.bg (K.G.); ivan.ginev@mu-plovdiv.bg (I.G.); 2 Department of Dentistry, Faculty of Dental Medicine, Medical University of Plovdiv, 4000 Plovdiv, Bulgaria; 21201016@mu-plovdiv.bg

**Keywords:** reconstructive surgery of the ear, temporalis muscle flap, external ear carcinoma, auricular reconstruction

## Abstract

**Background**: Auricular carcinoma is a malignant neoplasm arising from the epithelial or adnexal tissues of the external ear, most commonly representing cutaneous squamous cell carcinoma, basal cell carcinoma, or, less frequently, melanoma, characterized by early cartilage invasion and regional spread due to the thin soft-tissue envelope of the ear. Such malignancies involve radical resection, producing wide defects, which require complex reconstructive approach. **Case Presentation and Methods**: We describe a 45-year-old male patient who presented with a basal cell carcinoma, affecting the lobulus of the ear and the retroauricular and mastoid region. He underwent successful tumor resection with clear resection margins in an ENT department with a subsequent referral to our department for reconstruction. This report presents a successful reconstruction of a large post-excisional defect, 10 × 10 cm in diameter, with mastoid process exposure. Temporal muscle flip flap and skin graft, along with the preservation of the external auditory canal with Z-plasty, were incorporated for reconstruction after subtotal auricular amputation due to BCC. It highlights the importance of a diligent long-term follow-up, the preservation of the canal, preventing meatal stenosis, and the reconstructive potential of the muscle flap in patients with mastoid bone exposure in a single stage. **Conclusions**: A temporalis muscle flap with skin graft coverage offers a reliable, vascularized solution for large post-oncologic auricular defects while preserving the external auditory canal. Z-plasty remains a critical technique for preventing meatal stenosis.

## Simple Summary

Basal cell carcinoma affecting the external ear poses several challenges: It is classified as a high-risk cancer in the head and neck region, often requires radial and extensive resection, and reconstruction options are difficult to execute. We present a 45-year-old man with subtotal ear amputation, wide retroauricular resection with bone exposure, and reconstruction of the defect with a combined approach—Z-plasty to prevent meatal stenosis of the external auditory canal, temporalis muscle flip flap to cover the bone, and a skin graft to the muscle. This resection was performed after histological confirmation; 4-year follow-up revealed long-lasting results, no complications, and no recurrence of the disease.

## 1. Introduction

Cutaneous malignancies involving the auricle, such as basal cell carcinoma (BCC) and squamous cell carcinoma (SCC), are common due to chronic sun exposure, sun burns, repeated trauma, immunosuppression, and HPV infection. Skin neoplasms of the external ear often pose challenges because of the three-dimensional anatomy and topographic peculiarities of the auricle. Auricular skin cancers are more common in men and the helix is one of the most frequently affected sub-unit. Moreover, auricle malignancies are classified as part of the high-risk group of the head and neck region, which necessitates a radical surgical approach [1,2,3]. Achieving oncologically safe margins often requires subtotal or total auricular resection, creating complex composite defects.

Advanced auricular carcinomas involving the EAC and mastoid region can even require extensive resection in the retroauricular region in some cases. This surgical intervention often leaves severe exposure of the mastoid process, dead space around the EAC, and in cases in which chemo- or radiotherapy is included, the tissues remain poorly vascularized with higher risk for complications, such as infections, bone sequestration, and delayed healing. The research background poses the question why exposure of the mastoid bone and its management matter in the overall treatment protocol. Recent literature data emphasizes the covering of such defects with well-vascularized flaps to promote uneventful, faster healing and reduce the risk for infection and necrosis [4].

Reconstruction of such defects must provide stable coverage of exposed bone and cartilage, restore auricular contour, and ensure preservation of EAC to prevent stenosis.

A variety of techniques have been described, including local chondrocutaneous flaps, temporoparietal fascia flaps, temporalis muscle flap, free tissue transfer, and prosthetic reconstruction [1,2,3,4].

The temporalis muscle flap is a regional, pedicled option that can be rotated or flipped into the mastoid process and provides abundant vascularization and coverage, removing dead space and mitigating complications such as infection, bone sequestrum, and necrosis. A 2025 craniofacial review summarizes the temporal muscle flap’s advantages, such as dual vascular supply and volume thickness with low donor-site morbidity [5].

One recent literature example described a similar post-surgical defect, 8 × 7 cm in diameter, involving the EAC and bone, utilizing the temporal muscle flap and skin graft to close the defect. In this case, a silicone splint was used to preserve meatal patency [4].

To our knowledge, no prior report describes a one-stage reconstruction that combines a temporalis muscle flip flap with immediate skin grafting and meatal Z-plasty specifically to maintain EAC patency without the use of a stent in the setting of subtotal auriculectomy with mastoid exposure.

Unequivocally, the flap is a solid vascularized bed and a foundation for skin grafts and future autologous or prosthetic ear choices. Furthermore, the preservation of the EAC and hearing diversifies the subsequent pinna reconstruction methods.

## 2. Case Presentation

### 2.1. Patient History

**Figure 1:**
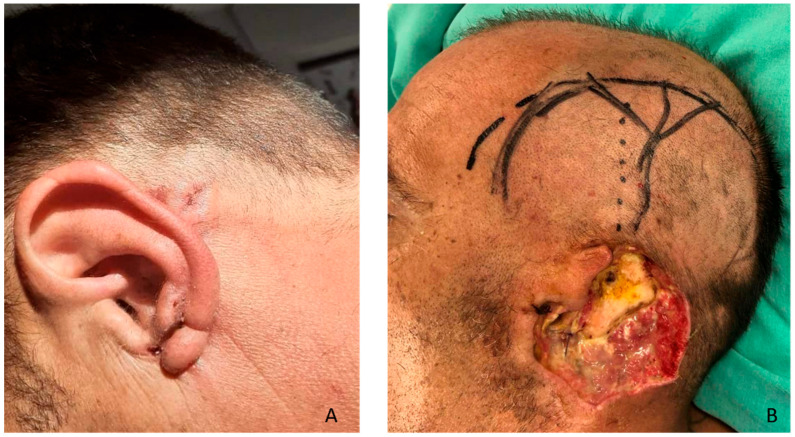
(**A**) Initial tumor lesion. (**B**) After radical excision with histologically verified clear resection margins. Temporalis muscle flip flap was designed to cover the large defect, including the exposed mastoid bone.

A 45-year-old man, without any comorbidities, presented with a history of a progressively enlarging ulcerated lesion of the left auricle and mastoid area for nearly 6–8 months (Figure 1A).

Biopsy confirmed BCC. Preoperative CT scan showed no spread to other organs. The staging of the disease is T3 N0 M0. Following multidisciplinary oncologic board discussion, the patient was referred for surgical management in the ENT department. A subtotal auricular resection was performed, along with partial (5 mm) excision of the EAC, and wide retroauricular resection, exposing the mastoid process, and leaving a large defect, 10 × 10 cm in diameter, as shown in Figure 1B.

### 2.2. Discussion of Surgical Approach

After histopathological verification of clear resection margins after two weeks, the patient was referred to our department for reconstruction of the large post-excisional defect. Surgical options were evaluated and the temporalis muscle flap with a skin graft from the inguinal area was utilized. Z-plasty for the EAC was considered to prevent stenosis. Other possibilities included free flaps, such as the ALT flap. However, the chosen surgical approach has significant advantages compared to free flaps, such as the shortened duration of surgery, lower risk for ICU admission, and lower burden of care.

### 2.3. Temporalis Muscle Flap—Design and Execution

**Figure 2:**
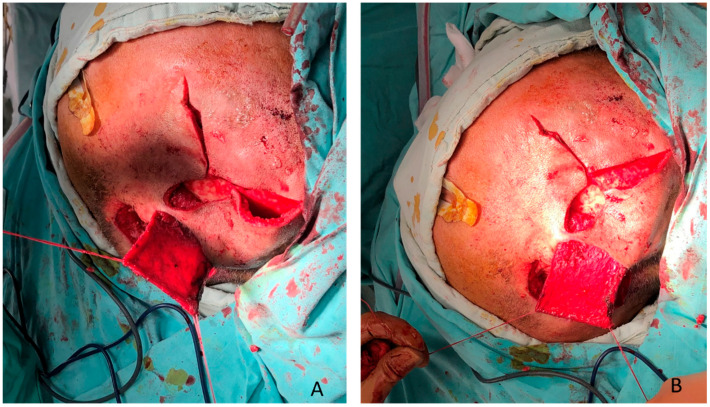
(**A**,**B**) Temporalis muscle flip flap covering the exposed bone.

A temporal muscle flap was designed, as dissection and elevation of the flap was carried out through a classical open temporal approach. The temporalis muscle was elevated from its origin along the temporal fossa using subperiosteal dissection to preserve the vascular pedicle. Posterior and superior attachments were released first, followed by careful detachment from the bone, while maintaining the integrity of the muscle and vascularity. Then, the flap was mobilized and flipped to provide vascularized coverage of the exposed mastoid bone, as shown in Figure 2.

### 2.4. Z-Plasty of the EAC and Skin Graft

**Figure 3:**
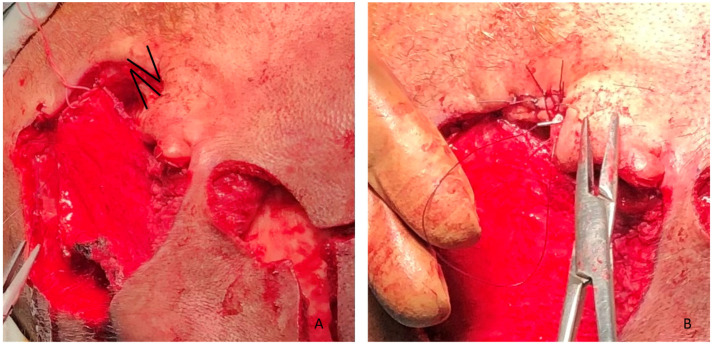
(**A**) Markings of the Z-plasty; (**B**) intraoperative result of Z-plasty.

**Figure 4:**
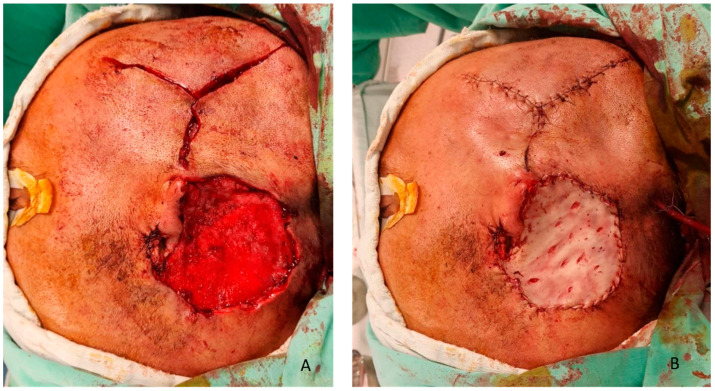
(**A**) Temporalis muscle flip flap covering the defect and Z-plasty of the external acoustic meatus; (**B**) skin graft to cover the muscle.

Z-plasty of the EAC was performed to preserve canal patency and prevent postoperative stenosis. Intraoperative markings can be observed in Figure 3. A manually perforated skin graft from the inguinal area was used as an external layer; see Figure 4.

### 2.5. Postoperative Period

The postoperative course was uneventful. Hospital stay was 3 days and therapy included 3 × 600 mg Clindamycin intravenously. Local dressings included bactigras gauze and additional antibiotic ointment. The flap demonstrated good perfusion, and at one-month follow-up, there was complete healing and full graft integration over the temporalis muscle flap.

### 2.6. Target Therapy

The patient was subsequently referred for targeted therapy with vismodegib (Erivedge) based on the tumor’s molecular profile and characteristics. Dosage of vismodegib was 150 mg once daily. However, due to experiencing side effects, the patient discontinued targeted therapy after 3 months of intake. No other adjuvant chemo- or radiotherapy was included in the treatment protocol. Regular postoperative imaging showed only fibrotic changes consistent with healing. The most recent MRI examination, four years after surgery, demonstrated no recurrence and a stable disease. The timespan for the next reconstructive stage was discussed with the patient and considered 5 years after the initial surgery, with MRI results showing no progression of the disease.

**Figure 5:**
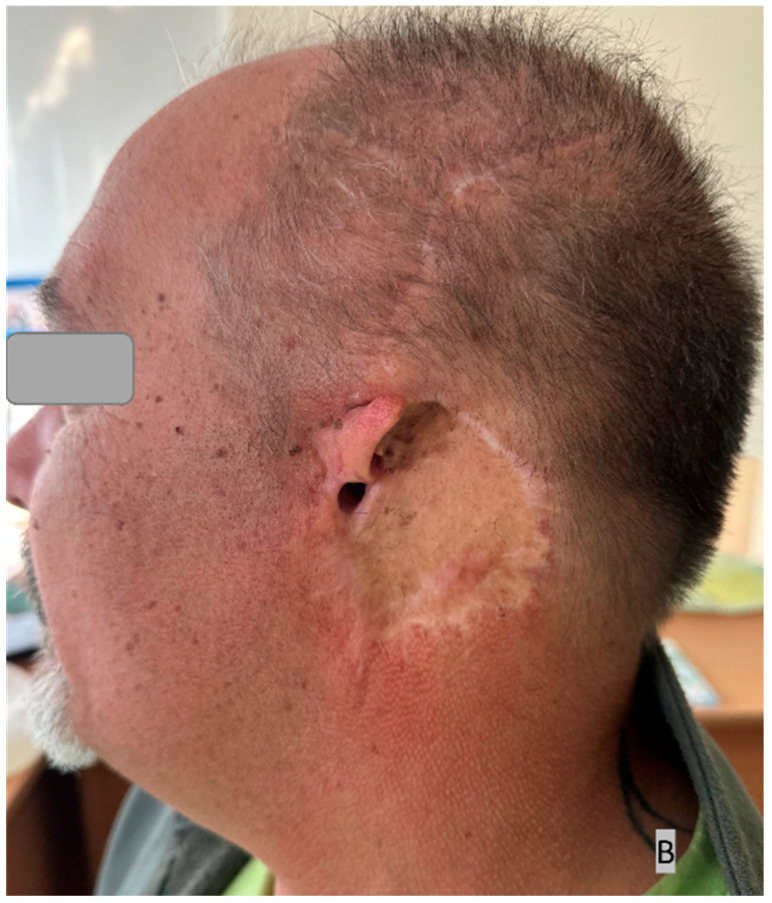
Results 4 years after surgery. No stenosis of the external meatus can be observed. The patient can be prepared for ear reconstruction.

Clinically, 4 years postoperatively, the patient exhibited a well-vascularized, stable reconstruction without meatal stenosis, suitable for future autologous cartilage or implant-based auricular reconstruction, as shown in Figure 5.

Other reconstructive options are novel technologies, such as 3D bioprinting, which are widely used in creating biocompatible implants, especially when the external ear is involved.

## 3. Discussion

Surgical management of complex oncological post-resection defects of the external ear and external acoustic meatus requires detailed understanding not only of the anatomical elements but also vascularity and innervation of local tissues. In our case, a subtotal ear amputation with exposure of the mastoid bone and disruption of the posterior wall of EAC, created a composite, multi-dimensional problem, involving skin, missing cartilage framework, and possible bone necrosis. Well-vascularized and thick coverage is required to reconstruct the area and prepare it for future outer ear reconstruction possibilities, including autologous cartilage, Medpor, etc. [6,7,8].

### 3.1. Anatomical Considerations and Reconstructive Implications

The auricle is an elastic cartilaginous framework, covered by thin skin and composed of the following subunits: helix, antihelix, concha, tragus, antitragus, and a fibrofatty subunit, called lobulus. The elastic cartilage of the auricle is covered by perichondrium, which is firmly attached to the anterior side and loosely to the posterior side. The auricle serves both a functional and aesthetic purpose, contributing to the hearing aspect and facial symmetry. Loss of the three-dimensional organ not only impairs the collection and directing of sound waves, but also causes severe cosmetic deformity. The EAC is critical for sound conduction and represents a surgically sensitive region due to its layered structural composition. From a reconstructive standpoint, the anatomical constraints of the EAC and the importance of maintaining the physiological diameter highlights the need for early, timely, and proactive stenosis prevention measures, especially following oncologic resections [1,6,7].

When the mastoid or temporal bone is exposed, a vascularized flap is mandatory to ensure durable coverage and minimize infection, necrosis, or sequestrum of the bone.

Reconstruction varieties depending on size of defect: A wide variety of techniques are available, chosen based on defect size, depth, patient factors, and surgeon preference. Options include:

Primary closure or healing by secondary intention: Small skin defects less than 1 cm may be closed primarily or left to heal spontaneously [9].

Skin grafting: Full-thickness or split-thickness skin grafts can cover non-critical areas or provide a skin layer over muscle flaps. Skin grafts alone cannot replace cartilage, but are useful for lining or superficial coverage of defects [9].

Local chondrocutaneous flaps: For moderate helix/lobule defects (1–3 cm), flap techniques like the Antia–Buch chondrocutaneous advancement, bilobed flaps, or transposition flaps from the adjacent auricle can reconstruct skin and cartilage in one stage. The Antia–Buch flap (and its modifications) is widely used for helical rim defects up to ~2 cm. Postauricular subcutaneous pedicled flaps can also be transposed to cover posterior ear or conchal defects [9].

Regional fascial and muscle flaps, such as the temporoparietal fascia flap (TPFF) and the temporalis muscle flap (TMF), are both well-vascularized, with proximity to local defects and minimal donor-site morbidity [10,11].

Free tissue transfer: For very large or composite defects, free flaps (e.g., radial forearm, anterolateral thigh, scapular, or perforator flaps) can bring well-vascularized skin and/or muscle from distant sites [12].

Cartilage framework and prosthesis: In cases of subtotal auriculectomy, reconstruction of the ear framework with autologous costal cartilage or alloplastic implants (e.g., porous polyethylene) can be staged once soft-tissue healing is complete. Alternatively, adhesive or osseointegrated auricular prostheses offer a non-surgical reconstruction choice [8,9].

Temporalis muscle flap—Anatomy and Advantages: The temporalis muscle flap (TMF) is a venerable and reliable choice for craniofacial reconstruction. The muscle originates from the temporal fossa of the skull and is involved in the coronoid process of the mandible [5]. The flap offers several advantages: robust and reliable vascularization, proximity to the defect, ability to cover exposed bone, and formation of a stable bed for secondary procedures such as cartilage framework placement or prosthetic fitting. It is an excellent regional flap for the reconstruction of head defects and it is widely recognized as a reliable and safe option for reconstruction after orbital exenteration, large midface defects, or facial palsy. It is classified under Mathes–Nahai type III flap with two dominant vascular pedicles—the deep temporal arteries (branches of the maxillary artery) and the middle temporal artery (branch of the superficial temporal artery). This type III Mathes–Nahai vascular pattern makes the TMF extremely robust even in irradiated or vessel-depleted fields [9,10,11].

### 3.2. Alternative Reconstruction Options

Alternatives to the temporalis muscle flap include the following:

The temporoparietal fascia flap (TPFF) is a thin, highly vascularized flap based on the superficial temporal artery. It is often harvested, pedicled, and tunneled to cover ear and scalp defects. The TPFF can be laid under a skin graft to create a conforming cover. It is noted in the literature that the TPFF is “thin, pliable, and well-vascularized” with “minimal donor morbidity,” making it a workhorse for auricular reconstruction [9,10]. Free flap transfer involves microvascular reconstructive surgery with a radial forearm free flap or an anterolateral (ALT) flap. These flaps provide sufficient coverage and vascularity. However, donor-site morbidity is higher compared to the usage of temporalis muscle flap. Secondly, survival is of greater risk when using the free flaps than with regional flaps. Thirdly, the use of free flaps significantly increases the duration of the surgery and it is associated with a higher rate of ICU admission. The recovery period and burden of care are significantly lower when employing regional head flaps instead of free flaps [12,13,14]. Cervicofacial advancement flap might be insufficient in widely exposed bone and scalp rotational flaps are more suitable for superiorly located defects. Other local transposition flaps, such as the bilobed flap, could be a possible option. However, for extensive defects with bone exposure, more robust vascularization and tissue volume is required to cover the defect [15,16,17,18]. Another alternative to the temporal flap is the supraclavicular island flap, which has a long pedicle and vast applications in various head and neck defects, providing sufficient coverage and relatively close texture and color match with the surrounding tissues [16].

### 3.3. EAC Stenosis Prevention

EAC stenosis prevention is a key point in the management of an advanced auricular cancer resection, a frequent complication after oncologic surgery in this region. Preservation of the canal lumen is important to mitigate the risk of conductive hearing loss. Surgeons may use techniques like Z-plasty or local skin advancement around the meatus to keep its patency [4,19]. For instance, in the presented case, a Z-plasty at the EAC was performed along with bone coverage with muscle flap elevation to avoid post-op stenosis. In other reports, temporary silicone stents have been used to maintain canal diameter during healing. Through careful flap design and execution, and mechanically enlarging the meatus with Z-plasty at the time of reconstruction, canal patency was maintained, eliminating the need for silicone splints or molds that are commonly required in the studies in the literature. The drawbacks of stenting the canal include infection, dislodge, prolonged maintenance, patient discomfort, and a potential delay in prosthetics planning [19].

### 3.4. Oncologic Considerations

Adjuvant therapy with vismodegib is consistent with current recommendations for high-risk, locally advanced, or recurrent BCC [20,21]. At four-year follow-up, the patient demonstrated stable reconstruction and no recurrence, supporting the durability and oncologic safety of this approach.

Failure to address all anatomical implications and local status may result in conductive hearing loss, recurrent infection, keratin debris accumulation, chronic otorrhea, and significantly low postoperative quality of life and functional outcomes.

At long-term follow-up, the patient maintained a stable reconstruction with no evidence of recurrence, confirming the durability and oncologic safety of this approach. Further reconstruction of the auricle with autologous tissues or implant is considered in the next stage of treatment.

## 4. Conclusions

Temporal muscle flip flap reconstruction, skin grafting, and meatal Z-plasty offers a safe, single-stage, and effective technique for the coverage of large post-oncologic defects involving the mastoid region. It ensures durable vascularized tissue, prevents meatal stenosis, and provides a solid foundation for secondary auricular reconstruction, if desired. Additionally, the temporal flap pertains several advantages over free or other regional flaps, concerning duration of surgery, rate of complications, and hospital stay. Furthermore, had the temporalis flap been unsuccessful, we would still have the opportunity to address the issue with the previously mentioned options. Although temporalis muscle flaps, skin grafts, and meatal plasties have each been described for peri-auricular and temporal bone reconstruction, they are usually applied in isolation or in staged procedures, and canal patency is typically maintained using silicone stents or molds. In contrast, our technique integrates a temporalis muscle flip flap, immediate skin grafting, and Z-plasty of the external auditory meatus in a single operation specifically designed to preserve canal patency, limiting potential complications, associated with stent usage. This combination addresses bone coverage, epithelialization, and anti-contracture mechanics simultaneously, resulting in a stable, patent canal and a prosthetic-ready periauricular surface. Long-term follow-up in this case confirms both oncologic control and functional success.

## Data Availability

The original contributions presented in this study are included in the article. Further inquiries can be directed to the corresponding author.

## Abbreviations

The following abbreviations are used in this manuscript:

BCC	Basal Cell Carcinoma
EAC	External auditory canal
EAM	External auditory meatus
SCC	Squamous cell carcinoma
HPV	Human Papilloma Virus
ENT	Ear, Nose, and Throat.
TMF	Temporalis Muscle Flap
